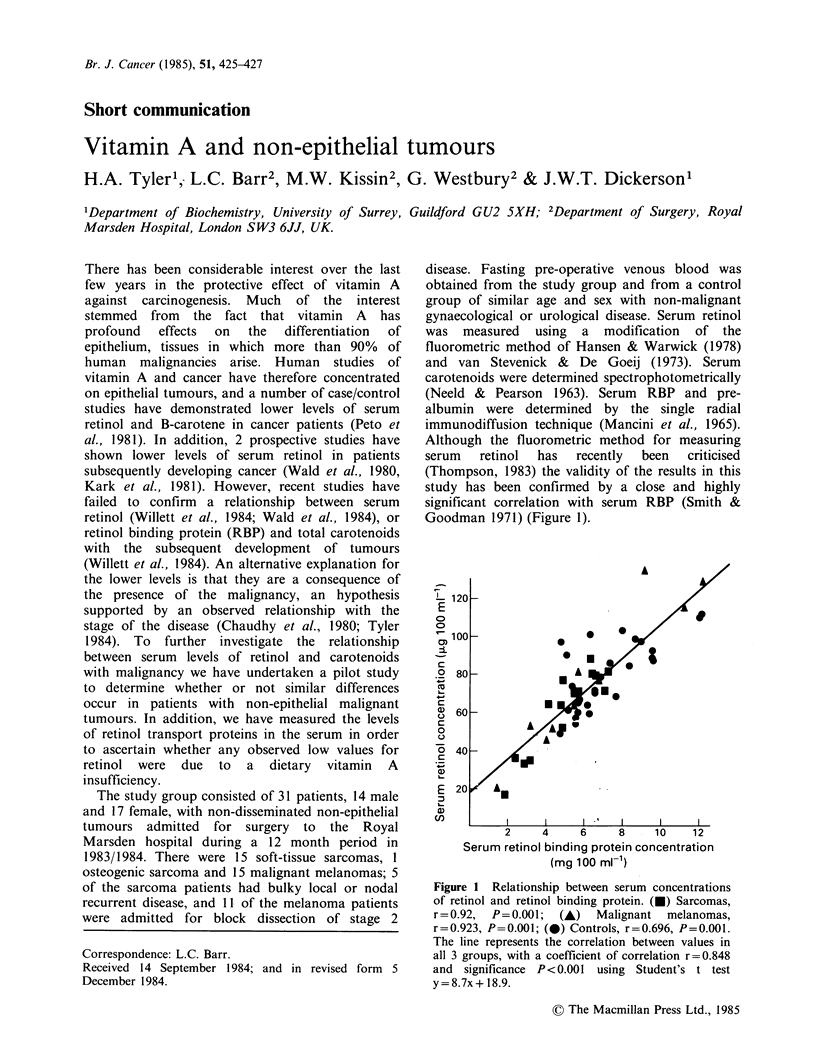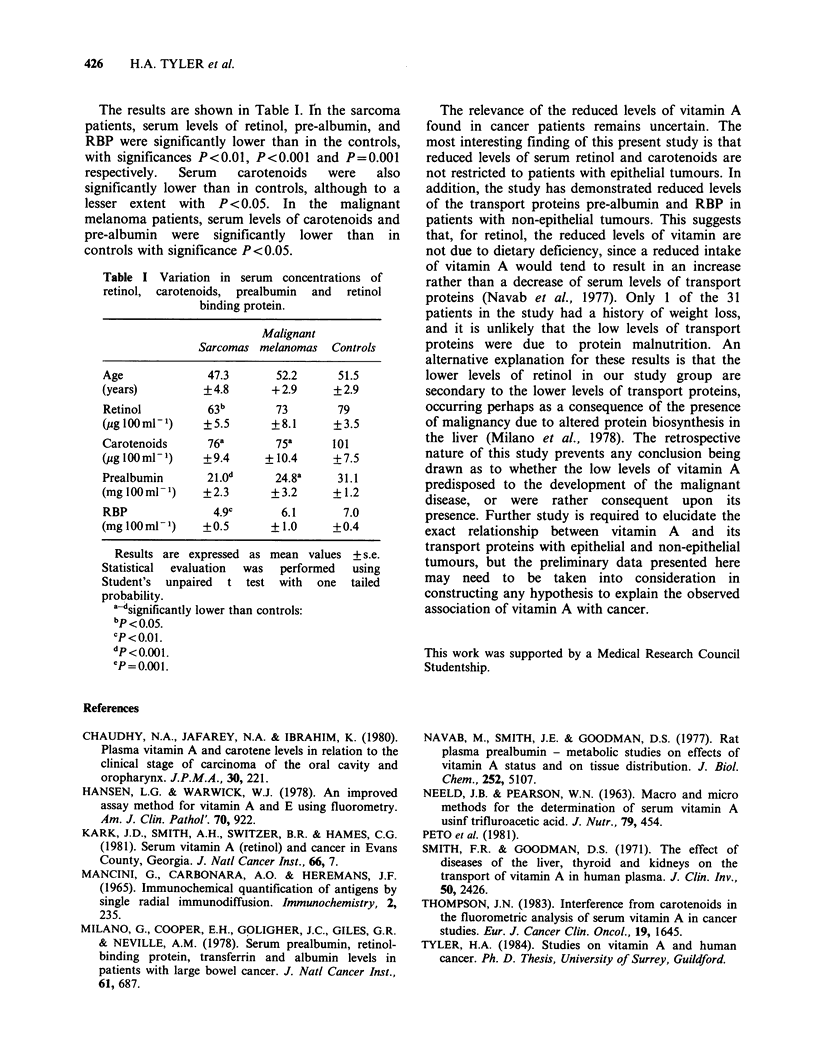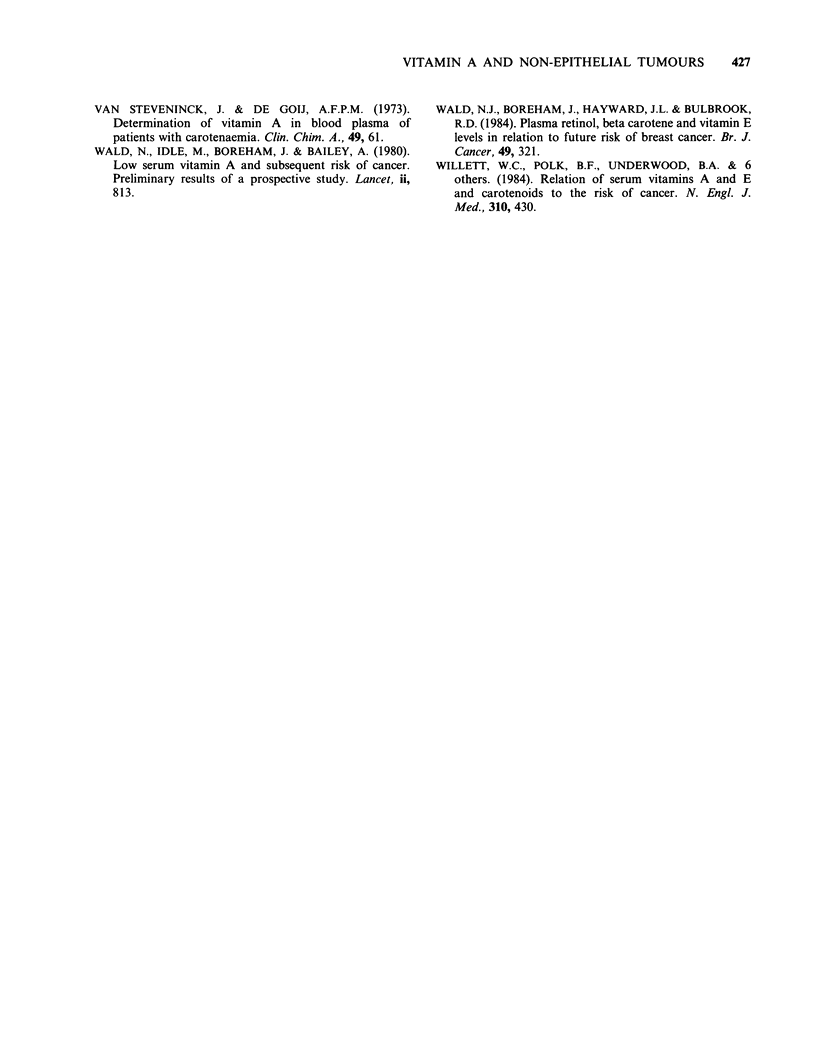# Vitamin A and non-epithelial tumours.

**DOI:** 10.1038/bjc.1985.58

**Published:** 1985-03

**Authors:** H. A. Tyler, L. C. Barr, M. W. Kissin, G. Westbury, J. W. Dickerson


*Br. J. Cancer* (1985), **51**, 425–427

### Short communication

### Vitamin A and non-epithelial tumours

H.A. Tyler[1], L.C. Barr[2], M.W. Kissin[2], G. Westbury[2] & J.W.T. Dickerson[1]

[1]*Department of Biochemistry, University of Surrey, Guildford GU2 5XH;* [2]*Department of Surgery, Royal Marsden Hospital, London SW3 6JJ, UK.*

There has been considerable interest over the last few years in the protective effect of vitamin A against carcinogenesis. Much of the interest stemmed from the fact that vitamin A has profound effects on the differentiation of epithelium, tissues in which more than 90% of human malignancies arise. Human studies of vitamin A and cancer have therefore concentrated on epithelial tumours, and a number of case/control studies have demonstrated lower levels of serum retinol and B-carotene in cancer patients (Peto *et al.,* 1981). In addition, 2 prospective studies have shown lower levels of serum retinol in patients subsequently developing cancer (Wald *et al.,* 1980, Kark *et al.,* 1981). However, recent studies have failed to confirm a relationship between serum retinol (Willett *et al.,* 1984; Wald *et al.,* 1984), or retinol binding protein (RBP) and total carotenoids with the subsequent development of tumours (Willett *et al.,* 1984). An alternative explanation for the lower levels is that they are a consequence of the presence of the malignancy, an hypothesis supported by an observed relationship with the stage of the disease (Chaudhy *et al.,* 1980; Tyler 1984). To further investigate the relationship between serum levels of retinol and carotenoids with malignancy we have undertaken a pilot study to determine whether or not similar differences occur in patients with non-epithelial malignant tumours. In addition, we have measured the levels of retinol transport proteins in the serum in order to ascertain whether any observed low values for retinol were due to a dietary vitamin A insufficiency.

The study group consisted of 31 patients, 14 male and 17 female, with non-disseminated non-epithelial tumours admitted for surgery to the Royal Marsden hospital during a 12 month period in 1983/1984. There were 15 soft-tissue sarcomas, 1 osteogenic sarcoma and 15 malignant melanomas; 5 of the sarcoma patients had bulky local or nodal recurrent disease, and 11 of the melanoma patients were admitted for block dissection of stage 2 disease. Fasting pre-operative venous blood was obtained from the study group and from a control group of similar age and sex with non-malignant gynaecological or urological disease. Serum retinol was measured using a modification of the fluorometric method of Hansen & Warwick (1978) and van Stevenick & De Goeij (1973). Serum carotenoids were determined spectrophotometrically (Neeld & Pearson 1963). Serum RBP and pre-albumin were determined by the single radial immunodiffusion technique (Mancini *et al.,* 1965). Although the fluorometric method for measuring serum retinol has recently been criticised (Thompson, 1983) the validity of the results in this study has been confirmed by a close and highly significant correlation with serum RBP (Smith & Goodman 1971) (Figure 1).

**Figure 1** Relationship between serum concentrations of retinol and retinol binding protein. (■) Sarcomas, $r=0.92$, $P=0.001$; (▲) Malignant melanomas, $r=0.923$, $P=0.001$; (●) Controls, $r=0.696$, $P=0.001$. The line represents the correlation between values in all 3 groups, with a coefficient of correlation $r=0.848$ and significance $P<0.001$ using Student's t test $y=8.7x+18.9$.

Correspondence: L.C. Barr.
Received 14 September 1984; and in revised form 5 December 1984.

© The Macmillan Press Ltd., 1985

The results are shown in Table I. In the sarcoma patients, serum levels of retinol, pre-albumin, and RBP were significantly lower than in the controls, with significances $P<0.01$, $P<0.001$ and $P=0.001$ respectively. Serum carotenoids were also significantly lower than in controls, although to a lesser extent with $P<0.05$. In the malignant melanoma patients, serum levels of carotenoids and pre-albumin were significantly lower than in controls with significance $P<0.05$.

**Table I** Variation in serum concentrations of retinol, carotenoids, prealbumin and retinol binding protein.

| | *Sarcomas* | *Malignant melanomas* | *Controls* |
|---|---|---|---|
| Age (years) | 47.3 ±4.8 | 52.2 +2.9 | 51.5 ±2.9 |
| Retinol ($\mu$g 100 ml$^{-1}$) | 63[b] ±5.5 | 73 ±8.1 | 79 ±3.5 |
| Carotenoids ($\mu$g 100 ml$^{-1}$) | 76[a] ±9.4 | 75[a] ±10.4 | 101 ±7.5 |
| Prealbumin (mg 100 ml$^{-1}$) | 21.0[d] ±2.3 | 24.8[a] ±3.2 | 31.1 ±1.2 |
| RBP (mg 100 ml$^{-1}$) | 4.9[c] ±0.5 | 6.1 ±1.0 | 7.0 ±0.4 |

Results are expressed as mean values ±s.e. Statistical evaluation was performed using Student's unpaired t test with one tailed probability.

[a–d]significantly lower than controls:

[b]$P<0.05$.

[c]$P<0.01$.

[d]$P<0.001$.

[e]$P=0.001$.

The relevance of the reduced levels of vitamin A found in cancer patients remains uncertain. The most interesting finding of this present study is that reduced levels of serum retinol and carotenoids are not restricted to patients with epithelial tumours. In addition, the study has demonstrated reduced levels of the transport proteins pre-albumin and RBP in patients with non-epithelial tumours. This suggests that, for retinol, the reduced levels of vitamin are not due to dietary deficiency, since a reduced intake of vitamin A would tend to result in an increase rather than a decrease of serum levels of transport proteins (Navab *et al.*, 1977). Only 1 of the 31 patients in the study had a history of weight loss, and it is unlikely that the low levels of transport proteins were due to protein malnutrition. An alternative explanation for these results is that the lower levels of retinol in our study group are secondary to the lower levels of transport proteins, occurring perhaps as a consequence of the presence of malignancy due to altered protein biosynthesis in the liver (Milano *et al.*, 1978). The retrospective nature of this study prevents any conclusion being drawn as to whether the low levels of vitamin A predisposed to the development of the malignant disease, or were rather consequent upon its presence. Further study is required to elucidate the exact relationship between vitamin A and its transport proteins with epithelial and non-epithelial tumours, but the preliminary data presented here may need to be taken into consideration in constructing any hypothesis to explain the observed association of vitamin A with cancer.

This work was supported by a Medical Research Council Studentship.

### References

CHAUDHY, N.A., JAFAREY, N.A. & IBRAHIM, K. (1980). Plasma vitamin A and carotene levels in relation to the clinical stage of carcinoma of the oral cavity and oropharynx. *J.P.M.A.*, **30**, 221.

HANSEN, L.G. & WARWICK, W.J. (1978). An improved assay method for vitamin A and E using fluorometry. *Am. J. Clin. Pathol'.* **70**, 922.

KARK, J.D., SMITH, A.H., SWITZER, B.R. & HAMES, C.G. (1981). Serum vitamin A (retinol) and cancer in Evans County, Georgia. *J. Natl Cancer Inst.*, **66**, 7.

MANCINI, G., CARBONARA, A.O. & HEREMANS, J.F. (1965). Immunochemical quantification of antigens by single radial immunodiffusion. *Immunochemistry*, **2**, 235.

MILANO, G., COOPER, E.H., GOLIGHER, J.C., GILES, G.R. & NEVILLE, A.M. (1978). Serum prealbumin, retinol-binding protein, transferrin and albumin levels in patients with large bowel cancer. *J. Natl Cancer Inst.*, **61**, 687.

NAVAB, M., SMITH, J.E. & GOODMAN, D.S. (1977). Rat plasma prealbumin – metabolic studies on effects of vitamin A status and on tissue distribution. *J. Biol. Chem.*, **252**, 5107.

NEELD, J.B. & PEARSON, W.N. (1963). Macro and micro methods for the determination of serum vitamin A usinf trifluroacetic acid. *J. Nutr.*, **79**, 454.

PETO *et al.* (1981).

SMITH, F.R. & GOODMAN, D.S. (1971). The effect of diseases of the liver, thyroid and kidneys on the transport of vitamin A in human plasma. *J. Clin. Inv.*, **50**, 2426.

THOMPSON, J.N. (1983). Interference from carotenoids in the fluorometric analysis of serum vitamin A in cancer studies. *Eur. J. Cancer Clin. Oncol.*, **19**, 1645.

TYLER, H.A. (1984). Studies on vitamin A and human cancer. *Ph. D. Thesis, University of Surrey, Guildford.*

VAN STEVENINCK, J. & DE GOIJ, A.F.P.M. (1973). Determination of vitamin A in blood plasma of patients with carotenaemia. *Clin. Chim. A.*, **49**, 61.

WALD, N., IDLE, M., BOREHAM, J. & BAILEY, A. (1980). Low serum vitamin A and subsequent risk of cancer. Preliminary results of a prospective study. *Lancet*, **ii**, 813.

WALD, N.J., BOREHAM, J., HAYWARD, J.L. & BULBROOK, R.D. (1984). Plasma retinol, beta carotene and vitamin E levels in relation to future risk of breast cancer. *Br. J. Cancer*, **49**, 321.

WILLETT, W.C., POLK, B.F., UNDERWOOD, B.A. & 6 others. (1984). Relation of serum vitamins A and E and carotenoids to the risk of cancer. *N. Engl. J. Med.*, **310**, 430.